# Supplementary material for: The Australian Injury Comorbidity Indices (AICIs) to predict in-hospital complications: A population-based data linkage study
Source: PLoS One. 2020 Sep 11;15(9):e0238182. doi: 10.1371/journal.pone.0238182 (PMC7485849; doi:10.1371/journal.pone.0238182)
Supplement: S1 Appendix — (DOCX) [file pone.0238182.s001.docx]

# Appendix A1 (SDC1): Residual plots

## ICU hours

Baseline model (age, sex, body region, injury type, injury severity geographic region)

Baseline + one comorbidity

Baseline + count of comorbidities

Baseline + presence of all 31 comorbidities

Baseline + Charlson Comorbidity Index (CCI)

Baseline + updated CCI

Baseline + Elixhauser Comorbidity Measure

Baseline + AICI-icu with binary representation of 5 comorbidities

Baseline + AICI-icu with actual weights (5 conditions)

Baseline + AICI-icu with rounded weights (5 conditions)

MV hours

Baseline (age, sex and injury severity)

Baseline + at least one comorbidity

Baseline + count of comorbidities

Baseline + all 31 comorbidities

Baseline + CCI

Baseline + updated CCI

Baseline + ECM

Baseline + AICI-mv with binary representation of 2 comorbidities

Baseline + AICI-mv with actual weights (1 condition)

Baseline + AICI-mv with rounded weights (1 condition)

## Complications

Baseline model (age, sex, body region, injury type and injury severity)

Baseline + one comorbidity

Baseline + count of comorbidities

Baseline + presence of all 31 comorbidities

Baseline + Charlson Comorbidity Index (CCI)

Baseline + updated CCI

Baseline + Elixhauser Comorbidity Measure

Baseline + AICI-comp with binary representation of 15 comorbidities

Baseline + AICI-comp with actual weights (5 conditions)

Baseline + AICI-comp with rounded weights (5 conditions)
