# Supplementary material for: The Australian Injury Comorbidity Indices (AICIs) to predict in-hospital complications: A population-based data linkage study
Source: PLoS One. 2020 Sep 11;15(9):e0238182. doi: 10.1371/journal.pone.0238182 (PMC7485849; doi:10.1371/journal.pone.0238182)
Supplement: S2 Appendix — (DOCX) [file pone.0238182.s002.docx]

# Appendix A2 (SDC2) – Interaction plots

## ICU hours

## MV hours

## Complications
