# Supplementary material for: The Australian Injury Comorbidity Indices (AICIs) to predict in-hospital complications: A population-based data linkage study
Source: PLoS One. 2020 Sep 11;15(9):e0238182. doi: 10.1371/journal.pone.0238182 (PMC7485849; doi:10.1371/journal.pone.0238182)
Supplement: S1 Table — (DOCX) [file pone.0238182.s003.docx]

A1 Table (SDC3.1): Performance of selected model fitting strategies in assessing the effect of comorbidity on selected outcome measures (Victoria)

| Model | Outcome | | | | | |
| --- | --- | --- | --- | --- | --- | --- |
|  | Ln (ICU hours)^1^ | | Ln (MV hours)^2^ | | Number of complications^3^ | |
|  | Adjusted R^2^ | Model fit AIC | Adjusted R^2^ | Model fit AIC | Mc.Fadden's Adjusted R^2^ | Model fit AIC |
| (viii) Baseline model + presence of at least one comorbidity | 0.110 | 14558 | 0.145 | 7817 | 0.032 | 106532 |
| (ix) Baseline model + count of comorbidities | 0.115 | 14530 | 0.145 | 7815 | 0.033 | 106461 |
| (x) Baseline model + individual comorbidity (all 31 conditions) | 0.125 | 14503 | 0.175 | 7758 | 0.036 | 106128 |
| (xi) Baseline model + selected comorbidities (modelled as a weighted summed score)^4^ | 0.121 | 14494 | 0.152 | 7795 | 0.032 | 106594 |
|  |  |  |  |  |  |  |
| Baseline model deconstructed |  |  |  |  |  |  |
| Age | 0.011 | 15073 | 0.027 | 8124 | 0.011 | 108842 |
| Age + Sex | 0.017 | 15047 | 0.032 | 8114 | 0.012 | 108799 |
| Age + Sex + Body region | 0.066 | 14788 |  |  | 0.015 | 108381 |
| Age + Sex + Body region + Injury type | 0.087 | 14686 |  |  | 0.024 | 107476 |
| Age + Sex + Body region + Injury type + Injury severity | 0.104 | 14591 | 0.145 | 7815 | 0.029 | 106848.6 |

Notes:

1. Baseline model includes age, sex, injury severity, injury type and body region; outcome =ICU stay hours (Ln transformed linear model)

2. Baseline model includes age, sex and injury severity; outcome= MV hours (Ln transformed linear model)

3. Baseline model includes age, sex, injury type, injury severity and body region; outcome=number of complications for those with at least one complication (negative binomial model)

4. Actual ORs used as weights, excludes weights resulting from an OR<1.2
