# Supplementary material for: The Australian Injury Comorbidity Indices (AICIs) to predict in-hospital complications: A population-based data linkage study
Source: PLoS One. 2020 Sep 11;15(9):e0238182. doi: 10.1371/journal.pone.0238182 (PMC7485849; doi:10.1371/journal.pone.0238182)
Supplement: S2 Table — (DOCX) [file pone.0238182.s004.docx]

A2 Table (SDC3.2): Performance of selected comorbidity measures in assessing the association between comorbidity and selected outcome measures (Victoria)

| Model | CHADx7^1^ (gastrointestinal complications) | | CHADx5^2^ (cardiovascular complications) | | CHADx15^3^ (metabolic disorders) | |
| --- | --- | --- | --- | --- | --- | --- |
|  | AUC (95% CI) | Model fit AIC | AUC (95% CI) | Model fit AIC | AUC (95% CI) | Model fit AIC |
| (i) Baseline model | 0.600 (0.593 to 0.608) | 31123 | 0.603 (0.595 to 0.610) | 29778 | 0.651 (0.643 to 0.658) | 27516 |
| (ii) Baseline model + selected comorbidities (individually modelled with binary representation) | 0.605 (0.598 to 0.613) | 31060 | 0.612 (0.605 to 0.620) | 29633 | 0.668 (0.661 to 0.675) | 27216 |
| (iv) Baseline model + comorbidity using CCI weights | 0.601 (0.594 to 0.609) | 31109 | 0.603 (0.595 to 0.611) | 29774 | 0.659 (0.652 to 0.666) | 27399 |
| (vi) Baseline model + ECM | 0.604 (0.597 to 0.611) | 31120 | 0.603 (0.595 to 0.610) | 29778 | 0.674 (0.667 to 0.681) | 27140 |

Notes:

1. Baseline model includes age, sex, injury type, body region and SEIFA; outcome= presence of CHADx7 (logistic model)

2. Baseline model includes age, sex, injury severity, injury type, body region and geographic region; outcome =presence of CHADx5 (logistic model)

3. Baseline model includes age, sex, injury type, injury severity, body region and geographic region; outcome= presence of CHADx15 (logistic model)

See Table A3 for selected comorbidities for each outcome
