# Supplementary material for: The Australian Injury Comorbidity Indices (AICIs) to predict in-hospital complications: A population-based data linkage study
Source: PLoS One. 2020 Sep 11;15(9):e0238182. doi: 10.1371/journal.pone.0238182 (PMC7485849; doi:10.1371/journal.pone.0238182)
Supplement: S3 Table — (DOCX) [file pone.0238182.s005.docx]

A3 Table (SDC3.3): Conditions included in the injury comorbidity indices for ICU stay, MV use, complications, CCI and ECM

| Comorbidity | Outcome | | | | | | | |
| --- | --- | --- | --- | --- | --- | --- | --- | --- |
|  | ICU hours (AICI-icu)^1^ | MV hours (AICI-mv)^2^ | Complications (AICI-comp)^3^ | Gastrointestinal complications | Cardiovascular complications | Metabolic disorders | CCI | ECM |
| HIV/AIDS | X | X | X | X | X | X | ✓ | ✓ |
| Alcohol dependence | X | ✓ | ✓ | X | X | ✓ | X | ✓ |
| Drug dependence | X | X | X | X | X | X | X | ✓ |
| Any malignancy | X | X | X | X | X | X | ✓^4^ | ✓^5^ |
| Blood loss anaemia | X | X | X | X | X | X | X | ✓ |
| Cardiac arrhythmias | X | X | ✓ | X | ✓ | ✓ | X | ✓ |
| Cerebrovascular disease | X | X | X | X | X | X | ✓ | X |
| Chronic pulmonary disease | ✓ | X | ✓ | X | ✓ | ✓ | ✓ | ✓ |
| Coagulopathy | ✓ | ✓ | ✓ | X | X | X | X | ✓ |
| Congestive heart failure | X | X | ✓ | X | ✓ | X | ✓ | ✓ |
| Deficiency anaemias | X | X | X | X | X | X | X | ✓ |
| Dementia | X | X | ✓ | ✓ | X | X | ✓ | X |
| Depression | ✓ | X | ✓ | X | X | X | X | ✓ |
| Diabetes with chronic complications | X | X | ✓ | X | X | X | ✓ | ✓ |
| Diabetes without complications | X | X | X | X | X | X | ✓ | ✓ |
| Fluid and electrolyte disorders | - | - | - | - | - | - | X | ✓ |
| Hemiplegia/paraplegia | X | X | X | X | X | X | ✓ | ✓ |
| Hypertension complicated | X | X | X | X | X | X | X | ✓ |
| Hypertension uncomplicated | ✓ | X | ✓ | X | ✓ | ✓ | X |  |
| Hypothyroidism | X | X | X | X | X | X | X | ✓ |
| Metastatic solid tumor | X | X | X | X | X | X | ✓ | ✓ |
| Mild liver disease | X | X | X | X | X | X | ✓ | ✓ |
| Moderate or severe liver disease | X | X | ✓ | X | X | ✓ | ✓ |  |
| Myocardial infarction | X | X | X | X | X | X | ✓ | X |
| Obesity | ✓ | X | ✓ | X | X | X | X | ✓ |
| Other neurological disorders | - | - | - | - | - | - | X | ✓ |
| Peptic ulcer disease | X | X | X | X | X | X | ✓ | ✓ |
| Peripheral vascular disease | X | X | ✓ | X | X | X | ✓ | ✓ |
| Psychoses | X | X | ✓ | X | X | X | X | ✓ |
| Pulmonary circulation disorders | X | X | X | X | X | X | X | ✓ |
| Renal disease including renal failure | X | X | ✓ | ✓ | X | ✓ | ✓ | ✓ |
| Rheumatic disease including some other connective tissue disorders | X | X | X | X | X | X | ✓ | ✓ |
| Valvular disease | X | X | ✓ | X | X | ✓ | X | ✓ |
| Weight loss | - | - | - | - | - | - | X | ✓ |

Notes:

1. AICI-icu - Australian Injury Comorbidity Index - ICU hours

2. AICI-mv - Australian Injury Comorbidity Index - MV hours

3. AICI-comp - Australian Injury Comorbidity Index -hospital acquired complications

4. Includes lymphoma, solid tumors without metastasis and leukaemia

5. Includes lymphoma and solid tumors without metastasis
