# Supplementary material for: The Australian Injury Comorbidity Indices (AICIs) to predict in-hospital complications: A population-based data linkage study
Source: PLoS One. 2020 Sep 11;15(9):e0238182. doi: 10.1371/journal.pone.0238182 (PMC7485849; doi:10.1371/journal.pone.0238182)
Supplement: S4 Table — (DOCX) [file pone.0238182.s006.docx]

A4 Table (SDC3.4): Performance of new comorbidity indices vs existing comorbidity indices in injury sub-groups (Victoria)

| Model | Ln (ICU hours) | | | | | |
| --- | --- | --- | --- | --- | --- | --- |
|  | Adults (25-64 years) | | Severe injury (adults) | | Intracranial injury^1^ (adults) | |
|  | Adjusted R2 | Model fit AIC | Adjusted R2 | Model fit AIC | Adjusted R2 | Model fit AIC |
| Baseline model^2^ | 0.134 | 7097 | 0.080 | 7826 | 0.111 | 1855 |
| Baseline model + AICI-icu | 0.157 | 7035 | 0.086 | 7813 | 0.129 | 1847 |
| Baseline model + comorbidity using CCI weights | 0.145 | 7068 | 0.080 | 7827 | 0.110 | 1856 |
| Baseline model + comorbidity using ECM | 0.180 | 6991 | 0.089 | 7831 | 0.114 | 1880 |
|  | Number of complications | | | | | |
|  | Older adults (>=65 years) | | Severe injury (adults) | | Intracranial injury^1^ (adults) | |
|  | Mc.Fadden's Adjusted R2 | Model fit AIC | Mc.Fadden's Adjusted R2 | Model fit AIC | Mc.Fadden's Adjusted R2 | Model fit AIC |
| Baseline model^3^ | 0.014 | 75988 | 0.004 | 52637.56 | 0.010 | 6333 |
| Baseline model + AICI-comp | 0.020 | 75511 | 0.010 | 52335.84 | 0.009 | 6335 |
| Baseline model + comorbidity using CCI weights | 0.015 | 75853 | 0.005 | 52589.38 | 0.009 | 6335 |
| Baseline model + comorbidity using ECM | 0.020 | 75502 | 0.009 | 52351.63 | 0.007 | 6350 |

Notes:

1. Intracranial injury = ICD-10-AM codes S06.00 - S06.9

2. Baseline model includes age, sex, injury severity, injury type and body region; outcome =ICU stay hours (ln transformed linear model)

3. Baseline model includes age, sex, injury severity, injury type and body region; outcome=grouped number of complications (negative binomial model)

A4 Table continued

| Model | Ln (ICU hours) | | | | | |
| --- | --- | --- | --- | --- | --- | --- |
|  | Hip-fracture^1^ | | Blunt trauma^2^ | | Penetrating trauma^3^ | |
|  | Adjusted R2 | Model fit AIC | Adjusted R2 | Model fit AIC | Adjusted R2 | Model fit AIC |
| Baseline model^4^ | 0.005 | 1761 | 0.103 | 8995 | 0.256 | 443 |
| Baseline model + AICI-icu | 0.004 | 1766 | 0.114 | 8963 | 0.251 | 448 |
| Baseline model + comorbidity using CCI weights | 0.004 | 1763 | 0.105 | 8988 | 0.251 | 445 |
| Baseline model + comorbidity using ECM | 0.046 | 1764 | 0.118 | 8976 | 0.243 | 460 |
|  | Number of complications | | | | | |
|  | Hip-fracture^1^ | | Blunt trauma^2^ | | Penetrating trauma^3^ | |
|  | Mc.Fadden's Adjusted R2 | Model fit AIC | Mc.Fadden's Adjusted R2 | Model fit AIC | Mc.Fadden's Adjusted R2 | Model fit AIC |
| Baseline model^5^ | 0.000 | 28490 | 0.026 | 92438 | 0.048 | 2331 |
| Baseline model + AICI-comp | 0.006 | 28296 | 0.033 | 91806 | 0.057 | 2305 |
| Baseline model + comorbidity using CCI weights | 0.001 | 28462 | 0.029 | 92224 | 0.048 | 2331 |
| Baseline model + comorbidity using ECM | 0.005 | 28327 | 0.033 | 91785 | 0.046 | 2317 |

Notes:

1. Hip fractures = ICD-10 codes S72.0 - S72.2, >= 45 years of age

2. Blunt trauma = ICD-10 codes V00-V99, W00-W19, W20-W24, W30-W31, W50-W52, X50, X79-X82, Y00-Y05 and Y29-Y32

3. Penetrating trauma = ICD-10 codes W53, W54, W55, W57, W58, W59, W25, W26, W27, W28, W29, W45, W32, W34, X72-X74, X78, X93-X95, X99, Y22-Y24 andY28

4. Baseline model includes age, sex, injury severity, injury type and body region; outcome =ICU stay hours (ln transformed linear model)

5. Baseline model includes age, sex, injury severity, injury type and body region; outcome=grouped number of complications (negative binomial model)
