# Supplementary material for: The Australian Injury Comorbidity Indices (AICIs) to predict in-hospital complications: A population-based data linkage study
Source: PLoS One. 2020 Sep 11;15(9):e0238182. doi: 10.1371/journal.pone.0238182 (PMC7485849; doi:10.1371/journal.pone.0238182)
Supplement: S5 Table — (DOCX) [file pone.0238182.s007.docx]

A5 Table (SDC3.5): Socio Economic Index for Areas (SEIFA) and country of birth details for the Victorian, NSW and WA study populations

|  | Patients admitted1, n (%) | | | | | | | | | | | |
| --- | --- | --- | --- | --- | --- | --- | --- | --- | --- | --- | --- | --- |
|  | July 2012 to June 2014, Victoria | | | | July 2012 to June 2014, NSW | | | | July 2012 to June 2014, WA | | | |
|  | n | % | At least one comorbidity (%) | Count of comorbidities, mean (95% CI) | n | % | At least one comorbidity (%) | Count of comorbidities, mean (95% CI) | n | % | At least one comorbidity (%) | Count of comorbidities, mean (95% CI) |
| Total patients | 161334 | | | | 233521 | | | | 84877 | | | |
|  |  |  |  |  |  |  |  |  |  |  |  |  |
| **SEIFA state decile^1^** | | | | | | | | | | | | |
| 1 | 17503 | 10.8 | 21.6 | 0.31 (0.30 to 0.32) | 17879 | 7.7 | 20.1 | 0.29 (0.28 to 0.30) | 1800 | 2.1 | 36.2 | 0.46 (0.43 to 0.49) |
| 2 | ** | ** | 20.3 | 0.28 (0.27 to 0.30) | 11249 | 4.8 | 19.7 | 0.29 (0.27 to 0.30) | 2359 | 2.8 | 25.5 | 0.33 (0.31 to 0.36) |
| 3 | 10595 | 6.5 | 22.0 | 0.32 (0.31 to 0.33) | 12527 | 5.4 | 22.4 | 0.32 (0.31 to 0.34) | 1813 | 2.1 | 22.0 | 0.29 (0.26 to 0.31) |
| 4 | 17975 | 11.1 | 21.1 | 0.29 (0.28 to 0.30) | 21142 | 9.1 | 21.6 | 0.31 (0.30 to 0.32) | 5713 | 6.7 | 22.7 | 0.31 (0.29 to 0.33) |
| 5 | 10601 | 6.6 | 17.6 | 0.25 (0.24 to 0.26) | 20382 | 8.7 | 18.3 | 0.26 (0.25 to 0.27) | 5656 | 6.7 | 21.4 | 0.29 (0.28 to 0.31) |
| 6 | 11830 | 7.3 | 18.8 | 0.27 (0.25 to 0.28) | 30030 | 12.9 | 18.7 | 0.27 (0.26 to 0.28) | 4759 | 5.6 | 20.5 | 0.29 (0.27 to 0.31) |
| 7 | 11678 | 7.3 | 20.0 | 0.28 (0.27 to 0.29) | 24255 | 10.4 | 18.9 | 0.27 (0.26 to 0.28) | 10226 | 12.0 | 18.2 | 0.25 (0.24 to 0.26) |
| 8 | 25726 | 16.0 | 18.9 | 0.26 (0.26 to 0.27) | 24339 | 10.4 | 17.6 | 0.25 (0.24 to 0.26) | 10719 | 12.6 | 20.8 | 0.29 (0.28 to 0.30) |
| 9 | 23873 | 14.8 | 19.6 | 0.27 (0.27 to 0.28) | 31664 | 13.6 | 19.2 | 0.28 (0.28 to 0.29) | 24734 | 29.1 | 19.3 | 0.26 (0.25 to 0.27) |
| 10 | 22902 | 14.2 | 18.7 | 0.26 (0.25 to 0.27) | 38996 | 16.7 | 16.9 | 0.25 (0.24 to 0.25) | 16777 | 19.8 | 19.2 | 0.26 (0.25 to 0.27) |
| 999 | * | * | * | * | 1058 | 0.5 | 33.9 | 0.45 (0.40 to 0.49) | 321 | 0.4 | 34.6 | 0.42 (0.35 to 0.50) |
|  |  |  |  |  |  |  |  |  |  |  |  |  |
| **Country of birth** | | | | | | | | | | | | |
| Australia | 121134 | 75.4 | 17.5 | 0.24 (0.24 to 0.24) | 178833 | 76.6 | 17.6 | 0.25 (0.25 to 0.25) | 60205 | 70.9 | 19.7 | 0.26 (0.26 to 0.27) |
| New Zealand | 2397 | 1.5 | 13.8 | 0.18 (0.16 to 0.20) | 4126 | 1.8 | 16.5 | 0.23 (0.21 to 0.25) | 3173 | 3.7 | 15.5 | 0.19 (0.17 to 0.21) |
| England | 4912 | 3.0 | 27.8 | 0.40 (0.38 to 0.42) | 8043 | 3.4 | 24.6 | 0.36 (0.34 to 0.37) | 6185 | 7.3 | 25.1 | 0.37 (0.35 to 0.39) |
| Italy | 3474 | 2.0 | 42.7 | 0.68 (0.65 to 0.71) | 2689 | 1.2 | 39.3 | 0.64 (0.60 to 0.67) | 1056 | 1.2 | 39.6 | 0.62 (0.56 to 0.68) |
| Greece | 2162 | 1.3 | 40.0 | 0.62 (0.58 to 0.66) | 1642 | 0.7 | 40.6 | 0.67 (0.62 to 0.72) | 147 | 0.2 | 44.9 | 0.72 (0.55 to 0.89) |
| India | 1657 | 1.1 | 16.4 | 0.22 (0.19 to 0.24) | 1607 | 0.7 | 15.6 | 0.22 (0.19 to 0.25) | 699 | 0.8 | 26.9 | 0.41 (0.35 to 0.48) |
| All other | 25598 | 15.8 | 25.0 | 0.36 (0.35 to 0.37) | 36581 | 15.7 | 22.7 | 0.34 (0.34 to 0.35) | 13412 | 15.8 | 20.4 | 0.29 (0.27 to 0.30) |

Notes:

1. Based on SLAs for Victoria and NSW, and LGAs for WA

*Cell count 1-4 suppressed to protect confidentiality

** Secondary cell suppression to maintain confidentiality of groups with 1-4 cases
