# Supplementary material for: The Australian Injury Comorbidity Indices (AICIs) to predict in-hospital complications: A population-based data linkage study
Source: PLoS One. 2020 Sep 11;15(9):e0238182. doi: 10.1371/journal.pone.0238182 (PMC7485849; doi:10.1371/journal.pone.0238182)
Supplement: S6 Table — (DOCX) [file pone.0238182.s008.docx]

A6 Table (SDC3.6): Presence of comorbidity with the mean LOS in the ICU and MV, and the mean number of complications (NSW and WA, age 15 years and over)

| Comorbidity | NSW | | | WA | | |
| --- | --- | --- | --- | --- | --- | --- |
|  | Index admissions (N=201791), n (%) | ICU stay hours for those using1 the ICU, mean (CI) | Complications for those with at least one, mean (CI) | Index admissions (N=71771), n (%) | ICU stay hours for those2 using the ICU, mean (CI) | Complications for those with at least one, mean (CI) |
| HIV/AIDS | 49 (0.0) | 81.3 (-44.2 to 206.7) | * | 24 (0.0) | * | * |
| Alcohol dependence | 10970 (5.4) | 76.7 (68.2 to 85.3) | 2.5 (2.4 to 2.7) | 6307 (8.8) | 71.5 (57.1 to 86.0) | 2.5 (2.3 to 2.8) |
| Drug dependence | 3108 (1.5) | 78.9 (63.8 to 94.0) | 2.5 (2.1 to 2.8) | 1347 (1.9) | 69.2 (45.9 to 92.5) | 1.9 (1.7 to 2.2) |
| Any malignancy | 846 (0.4) | 57.5 (45.8 to 69.3) | 3.4 (3.0 to 3.8) | 307 (0.4) | 175.6 (-80.2 to 431.5) | 3.0 (2.5 to 3.4) |
| Blood loss anaemia | 152 (0.1) | 91.2 (44.9 to 137.5) | 3.4 (2.7 to 4.0) | 105 (0.2) | 148.2 (24.8 to 271.6) | 3.1 (2.4 to 3.8) |
| Cardiac arrhythmias | 5588 (2.8) | 111.6 (98.9 to 124.2) | 3.5 (3.3 to 3.6) | 1516 (2.1) | 164.4 (111.4 to 217.3) | 3.6 (3.3 to 3.8) |
| Cerebrovascular disease | 1078 (0.5) | 150.4 (109.3 to 191.5) | 3.5 (3.2 to 3.8) | 352 (0.5) | 191.8 (103.1 to 280.5) | 3.2 (2.7 to 3.6) |
| Chronic pulmonary disease | 1676 (0.8) | 90.6 (72.3 to 108.8) | 3.3 (3.1 to 3.6) | 442 (0.6) | 98.4 (28.0 to 168.8) | 3.3 (2.9 to 3.7) |
| Coagulopathy | 1225 (0.6) | 164.6 (128.4 to 200.8) | 3.7 (3.4 to 4.1) | 517 (0.7) | 251.8 (135.0 to 368.6) | 3.8 (3.3 to 4.2) |
| Congestive heart failure | 1407 (0.7) | 95.7 (79.6 to 111.7) | 3.7 (3.5 to 3.9) | 429 (0.6) | 100.8 (49.8 to 151.8) | 4.4 (3.9 to 4.9) |
| Deficiency anaemias | 683 (0.3) | 54.1 (34.8 to 73.4) | 3.4 (2.9 to 3.8) | 216 (0.3) | 294.2 (-148.8 to 737.1) | 3.1 (2.5 to 3.8) |
| Dementia | 5694 (2.8) | 65.2 (49.6 to 80.8) | 2.8 (2.7 to 2.9) | 1534 (2.1) | 67.6 (31.5 to 103.8) | 2.8 (2.6 to 3.0) |
| Depression | 5713 (2.8) | 56.5 (51.0 to 62.0) | 2.6 (2.3 to 2.8) | 1266 (1.8) | 110.1 (52.3 to 168.0) | 2.6 (2.3 to 3.0) |
| Diabetes with chronic complications | 4466 (2.2) | 105.0 (84.9 to 125.1) | 3.4 (3.2 to 3.6) | 1726 (2.4) | 173.8 (99.6 to 248.0) | 3.2 (3.0 to 3.5) |
| Diabetes without complications | 10717 (5.3) | 90.7 (76.9 to 104.4) | 2.9 (2.8 to 3.0) | 4349 (6.1) | 132.9 (79.5 to 186.2) | 2.7 (2.5 to 2.9) |
| Hemiplegia/paraplegia | 962 (0.5) | 194.9 (142.3 to 247.4) | 3.5 (3.1 to 3.9) | 338 (0.5) | 146.4 (78.6 to 214.3) | 3.6 (2.9 to 4.2) |
| Hypertension complicated | 44 (0.0) | * | 4.1 (2.7 to 5.4) | 29 (0.0) | * | 2.8 (2.0 to 3.7) |
| Hypertension uncomplicated | 7057 (3.5) | 113.0 (100.5 to 125.5) | 3.4 (3.3 to 3.6) | 2077 (2.9) | 198.0 (138.2 to 257.8) | 3.6 (3.4 to 3.8) |
| Hypothyroidism | 312 (0.2) | 139.0 (-0.6 to 278.6) | 3.0 (2.4 to 3.6) | 73 (0.1) | * | 3.4 (2.1 to 4.7) |
| Metastatic solid tumor | 477 (0.2) | 68.3 (44.9 to 91.7) | 3.6 (2.9 to 4.2) | 157 (0.2) | 230.4 (-247.6 to 708.4) | 3.1 (2.5 to 3.7) |
| Mild liver disease | 1351 (0.7) | 113.4 (91.1 to 135.7) | 3.3 (2.9 to 3.7) | 550 (0.8) | 91.7 (54.9 to 128.6) | 3.1 (2.5 to 3.7) |
| Moderate or severe liver disease | 159 (0.1) | 168.4 (80.0 to 256.9) | 3.5 (2.8 to 4.2) | 55 (0.1) | 114.4 (61.5 to 167.3) | 4.1 (2.7 to 5.5) |
| Myocardial infarction | 455 (0.2) | 126.9 (59.2 to 194.7) | 3.8 (3.3 to 4.4) | 126 (0.2) | * | 4.1 (3.2 to 4.9) |
| Obesity | 501 (0.3) | 160.7 (101.2 to 220.2) | 3.9 (3.2 to 4.5) | 140 (0.2) | 295.8 (-25.0 to 616.5) | 4.4 (3.1 to 5.7) |
| Peptic ulcer disease | 105 (0.1) | 90.4 (48.1 to 132.8) | 4.1 (2.9 to 5.2) | 55 (0.1) | 65.2 (16.5 to 113.9) | 4.2 (2.3 to 6.0) |
| Peripheral vascular disease | 447 (0.2) | 207.7 (100.4 to 314.9) | 3.7 (3.1 to 4.2) | 391 (0.5) | 85.4 (31.1 to 139.6) | 3.1 (2.6 to 3.6) |
| Psychoses | 937 (0.5) | 88.6 (67.3 to 110.0) | 2.8 (2.4 to 3.3) | 182 (0.3) | 106.9 (47.5 to 166.4) | 3.6 (2.1 to 5.0) |
| Pulmonary circulation disorders | 349 (0.2) | 123.5 (84.3 to 162.7) | 3.7 (3.3 to 4.2) | 91 (0.1) | 358.2 (-66.9 to 783.3) | 4.3 (3.0 to 5.6) |
| Renal disease including renal failure | 3457 (1.7) | 98.0 (76.8 to 119.1) | 3.6 (3.5 to 3.8) | 1095 (1.5) | 163.1 (80.6 to 245.6) | 3.4 (3.1 to 3.8) |
| Rheumatic disease including some other connective tissue disorders | 307 (0.2) | 96.4 (38.3 to 154.4) | 2.9 (2.4 to 3.3) | 107 (0.2) | 0.0 (0.0 to 0.0) | 3.8 (2.8 to 4.8) |
| Valvular disease | 551 (0.3) | 112.4 (75.9 to 149.0) | 3.6 (3.2 to 4.0) | 159 (0.2) | 185.8 (-28.4 to 400.0) | 4.3 (3.5 to 5.1) |

Notes:

*Cell count 1-4 suppressed to protect confidentiality

1. n=6696

2. n=713
