# Supplementary material for: The Australian Injury Comorbidity Indices (AICIs) to predict in-hospital complications: A population-based data linkage study
Source: PLoS One. 2020 Sep 11;15(9):e0238182. doi: 10.1371/journal.pone.0238182 (PMC7485849; doi:10.1371/journal.pone.0238182)
Supplement: S7 Table — (DOCX) [file pone.0238182.s009.docx]

A7 Table (SDC3.7): Performance of selected model fitting strategies in assessing the effect of comorbidity on selected outcome measures (NSW and WA)

| Models | Ln (ICU hours) | | Number of complications | |
| --- | --- | --- | --- | --- |
|  | Adjusted R^2^ | Model fit AIC | McFadden's Adjusted R^2^ | Model fit AIC |
| NSW |  |  |  |  |
| (i) Baseline model^1,2^ | 0.065 | 22058 | 0.019 | 64933 |
| (ii) Baseline model + individual comorbidity (selected) (binary representation) | 0.070 | 22021 | 0.023 | 64619 |
| (iii) Baseline model + comorbidity using ICI (integer value of actual weight) | 0.069 | 22025 | 0.020 | 64847 |
| (iv) Baseline model + comorbidity using CCI weights | 0.066 | 22048 | 0.021 | 64746 |
| (v) Baseline model + comorbidity using Quan weights | 0.067 | 22042 | 0.020 | 64818 |
| (vi) Baseline model + ECM | 0.082 | 21962 | 0.024 | 64575 |
|  |  |  |  |  |
| WA |  |  |  |  |
| (i) Baseline model^1,2^ | 0.189 | 2209 | 0.029 | 29387 |
| (ii) Baseline model + individual comorbidity (selected) (binary representation) | 0.230 | 2177 | 0.037 | 29148 |
| (iii) Baseline model + comorbidity using ICI (integer value of actual weight) | 0.225 | 2178 | 0.032 | 29296 |
| (iv) Baseline model + comorbidity using CCI weights | 0.200 | 2200 | 0.032 | 29317 |
| (v) Baseline model + comorbidity using Quan weights | 0.194 | 2206 | 0.031 | 29329 |
| (vi) Baseline model + ECM | 0.238 | 2193 | 0.038 | 29125 |

Notes:

1. Baseline model includes age, sex, injury severity, injury type and body region; outcome =ICU stay hours (Ln transformed linear model)

2. Baseline model includes age, sex, injury type, injury severity and body region; outcome=number of complications for those with at least one complication (negative binomial model)

note: See Table A3 for selected comorbidities for each outcome
